# Supplementary material for: Simple and Effective Derivatization of Amino Acids with 1-Fluoro-2-nitro-4-(trifluoromethyl)benzene in a Microwave Reactor for Determination of Free Amino Acids in Kombucha Beverages
Source: Materials (Basel). 2022 Oct 20;15(20):7365. doi: 10.3390/ma15207365 (PMC9611567; doi:10.3390/ma15207365)
Supplement: Supplementary file 1 [file materials-15-07365-s001.zip › materials-1935445-supplementary.pdf]

Article

Simple and Effective Derivatization of Amino Acids with 1-Fluoro-2-nitro-4-(trifluoromethyl)benzene in a Microwave Reactor for Determination of Free Amino Acids in Kombucha Beverages

Aneta Jastrzębska [1,*], Zuzanna Gralak [1], Kamil Brzuzy [1], Anna Kmieciak [2], Marek P. Krzemiński [2], Rafał Burdziński [1], Marzanna Kurzawa [1] and Edward Szłyk [1]

[1] Department of Analytical Chemistry and Applied Spectroscopy, Faculty of Chemistry, Nicolaus Copernicus University in Toruń, Gagarin 7 Str., 87-100 Toruń, Poland
[2] Department of Organic Chemistry, Faculty of Chemistry, Nicolaus Copernicus University in Toruń, Gagarin 7 Str., 87-100 Toruń, Poland
* Correspondence: aj@umk.pl

Table S1. The reaction yields between FNBT and amino acids under tested conditions

	Trp	Lys	Phe	His
AA, FNBT, ACN, pH=9	99.9	93.7	98.4	91.5
AA, FNBT, pH=9	99.8	93.7	98.1	91.3
AA, FNBT, ACN, pH=10	100.0	99.4	100.0	99.6
AA, FNBT, pH=10	100.0	99.4	100.0	99.5

Where: AA - amino acid; FNBT – 4-Fluoro-3-nitrobenzotrifluoride; ACN – acetonitrile; Trp – Tryptophan, Lys- Lysine, Phe – Phenylalanine; His - Histidine

Ala-FNBT

(2-nitro-4-(trifluoromethyl)phenyl)-L-alanine

mass of product: 409 mg, Yield: 97.99%, mp. 123-125°C, $[\alpha]_D^{27}$ 9.46 (C 0.976, MeOH)

^{1}H NMR (700 MHz, DEUTERIUM OXIDE) Shift ppm 2.41 (d, J=6.67 Hz, 3 H), 3.49 (m, 1 H), 6.99 (m, 1 H), 7.57 (m, 1 H), 8.28 (m, 1 H)

^{13}C NMR (176 MHz, DEUTERIUM OXIDE) Shift ppm, 33.46, 39.70, 115.15, 116.62 (q, J=34.47 Hz), 122.94 (q, J=269.71 Hz), 124.71, 129.97, 132.42, 147.05, 180.39

^{19}F NMR (376 MHz, DEUTERIUM OXIDE) Shift ppm -62.80

Cys-FNBT

(2-nitro-4-(trifluoromethyl)phenyl)-L-cysteine

mass of product: 439 mg, Yield: 94.32%, oil, $[\alpha]_D^{27}$ 798,79 (C 0,964, MeOH)

^{1}H NMR (700 MHz, DEUTERIUM OXIDE) Shift ppm 3.34-3.40 (dd, J=7.24, 2 H), 4.00-4.09 (m, 1 H), 6.85 (m, 1 H), 7.65 (m, 1 H), 8.46 (m, 1 H)

^{13}C NMR (176 MHz, DEUTERIUM OXIDE) Shift ppm 24.88, 62.17, 115.38, 116.84 (q, J=33.91 Hz), 123.77 (q, J=269.73 Hz), 124.88, 130.30, 132.65, 146.61, 179.94

^{19}F NMR (376 MHz, DEUTERIUM OXIDE) Shift ppm –62.69

Asp-FNBT

(2-nitro-4-(trifluoromethyl)phenyl)-L-aspartic acid

mass of product: 455 mg, Yield: 94.20%, mp. 164-171°C, $[\alpha]_D^{27}$ 6.92 (C 0.976, MeOH)

^{1}H NMR (700 MHz, DEUTERIUM OXIDE) Shift ppm 2.55-2.60 (m, 1 H), 2.77 (dd, J=14.94 Hz, J=3.87 Hz, 1 H), 4.31 (dd, J=9.60, J=4.00 Hz, 1H), 6.84 (m, 1 H), 7.63 (m, 1 H), 8.40 (m, 1 H)

^{13}C NMR (176 MHz, DEUTERIUM OXIDE) Shift ppm 40.61, 56.40, 115.53, 116.87 (q, J=33.13 Hz), 124.85 (q, J=270.34 Hz), 124.88, 130.54, 132.58, 146.53, 178.72, 178.87

^{19}F NMR (376 MHz, DEUTERIUM OXIDE) Shift ppm -62.77

Glu-FNBT

(2-nitro-4-(trifluoromethyl)phenyl)-L-glutamic acid

mass of product: 474 mg, Yield: 93.98%, mp. 165-169°C, $[\alpha]_D^{26}$ -47.87 (C 0.986, MeOH)

^{1}H NMR (700 MHz, DEUTERIUM OXIDE) Shift ppm 1.99-2.06 (m, 2 H), 2.22-2.26 (m, 2 H), 4.05-4.10 (m, 1H), 6.86 (m, 1 H), 7.63 (m, 1 H), 8.40 (m, 1 H)

^{13}C NMR (176 MHz, DEUTERIUM OXIDE) Shift ppm 28.74, 33.72, 58.11, 115.47, 116.81 (q, J=34.13 Hz), 124.57 (q, J=269.71 Hz), 124.94, 130.51, 132.59, 146.46, 178.93, 182.06

^{19}F NMR (376 MHz, DEUTERIUM OXIDE) Shift ppm –61.27

Phe-FNBT

(2-nitro-4-(trifluoromethyl)phenyl)-L-phenylalanine

mass of product: 531 mg, Yield: 100%, mp. 253-255°C, $[\alpha]_D^{27}$ -91.84 (C 0.964, MeOH)

^{1}H NMR (700 MHz, DEUTERIUM OXIDE) Shift ppm 2.94 (dd, J=13.77 Hz, J=7.53 Hz, 1 H), 3.11 (dd, J=13.98 Hz, J=4.73 Hz, 1 H), 4.14 (dd, J=7.20 Hz, J=4.80 Hz, 1 H) 6.46 (m, 1 H), 7.02-7.08 (m, 3 H), 7.08-7.13 (m, 2 H), 7.22 (m, 1 H), 8.03 (m, 1 H)

^{13}C NMR (176 MHz, DEUTERIUM OXIDE) Shift ppm 37.91, 59.33, 115.29, 116.67 (q, J=34.80 Hz), 124.29 (q, J=269.71 Hz), 124.48, 126.98, 127.66, 128.54, 129.96, 132.21, 136.90, 146.95, 177.96

^{19}F NMR (376 MHz, DEUTERIUM OXIDE) Shift ppm –61.36

Gly-FNBT

(2-nitro-4-(trifluoromethyl)phenyl)-L-glycine

mass of product: 389 mg, Yield: 98.14%, mp. 108-110°C, $[\alpha]_D^{27}$ 2.70 (C 0.988, MeOH)

^{1}H NMR (700 MHz, DEUTERIUM OXIDE) Shift ppm 3.87 (s, 2 H), 6.76 (m, 1 H), 7.59 (m, 1 H), 8.33 (m, 1 H)

^{13}C NMR (176 MHz, DEUTERIUM OXIDE) Shift ppm 46.37, 115.29, 116.65 (q, J=33.46 Hz), 123.74 (q, J=270.04 Hz), 124.96, 130.13, 132.41, 146.68, 176.54

^{19}F NMR (376 MHz, DEUTERIUM OXIDE) Shift ppm -62.79

His-FNBT

(2-nitro-4-(trifluoromethyl)phenyl)-L-histidine

mass of product: 510 mg, Yield: 98.68%, mp. 177-178°C, $[\alpha]_D^{27}$ -55.19 (C 0.976, MeOH)

^{1}H NMR (700 MHz, DEUTERIUM OXIDE) Shift ppm 3.21-3.25 (m, 1 H), 3.25-3.30 (m, 1 H), 4.40 (m, 1 H), 6.84 (m, 1 H), 7.11 (s, 1 H), 7.61 (m, 1 H), 8.22 (s, 1 H), 8.35 (m, 1 H)

^{13}C NMR (176 MHz, DEUTERIUM OXIDE) Shift ppm 27.16, 64.34, 115.26, 116.35 (q, J=34.06 Hz), 117.98, 123.64 (q, J=270.00 Hz), 124.91, 130.17, 132.51, 136.23, 146.88, 177.34

^{19}F NMR (376 MHz, DEUTERIUM OXIDE) Shift ppm –61.27

Ile-FNBT

(2-nitro-4-(trifluoromethyl)phenyl)-L-isoleucine

mass of product: 480 mg, Yield: 99.85%, mp. 76-79°C, $[\alpha]_D^{27}$ 6.29 (C 1.012, MeOH)

^{1}H NMR (700 MHz, DEUTERIUM OXIDE) Shift ppm 0.84 (t, J=7.40 Hz, 3 H), 0.89 (d, J=6.94 Hz, 3 H), 1.19-1.23 (m, 1 H), 1.47-1.51 (m, 1 H), 1.83-1.91 (m, 1 H), 3.88 (d, J=5.47 Hz, 1 H), 6.77 (m, 1 H), 7.54 (m, 1 H), 8.28 (m, 1 H)

^{13}C NMR (176 MHz, DEUTERIUM OXIDE) Shift ppm 10.95, 15.38, 25.14, 37.33, 63.12, 115.34, 116.56 (q, J=34.13 Hz), 123.68 (q, J=270.04 Hz), 124.73, 130.05, 132.53, 146.78, 178.60

^{19}F NMR (376 MHz, DEUTERIUM OXIDE) Shift ppm -62.85

Leu-FNBT

(2-nitro-4-(trifluoromethyl)phenyl)-L-leucine

mass of product: 478 mg, Yield: 99.41%, oil, $[\alpha]_D^{27}$ 0.99 (C 0.972, MeOH)

^{1}H NMR (700 MHz, DEUTERIUM OXIDE) Shift ppm 0.81 (d, J=6.00 Hz, 3 H), 0.88 (d, J=6.14 Hz, 3 H), 1.59-1.63 (m, 1 H), 1.65-1.74 (m, 2 H), 4.01 (dd, J=8.02 Hz, J=4.87 Hz, 1 H), 6.80 (m, 1 H), 7.60 (m, 1 H), 8.35 (m, 1 H)

^{13}C NMR (176 MHz, DEUTERIUM OXIDE) Shift ppm 21.22, 22.30, 24.81, 41.30, 57.27, 115.28, 116.81 (q, J=34.47 Hz), 123.53 (q, J=269.37 Hz), 124.84, 130.26, 132.64, 146.56, 180.22

^{19}F NMR (376 MHz, DEUTERIUM OXIDE) Shift ppm -62.82

Lys-FNBT

N^2,N^6-bis(2-nitro-4-(trifluoromethyl)phenyl)-L-lysine

mass of product: 774 mg, Yield: 98.46%, oil, $[\alpha]_D^{27}$ -34.23 (C 0.970, MeOH)

^{1}H NMR (400 MHz, DEUTERIUM OXIDE) Shift ppm 1.56-1.70 (m, 6 H), 4.06-4.09 (m, 1 H), 4.22-4.26 (m, 2 H), 6.64 (m, 1 H), 6.66 (m, 1 H), 7.68 (m, 1 H), 7.71 (m, 1 H), 8.32-8.38 (m, 2 H)

^{13}C NMR (176 MHz, DEUTERIUM OXIDE) Shift ppm 23.34, 30.78, 31.43, 49.37, 68.53, 115.29, 115.54, 116.65 (q, J=33.46 Hz), 116.90 (q, J=31.45 Hz), 123.56 (q, J=268.88 Hz), 123.74 (q, J=270.04 Hz), 124.96, 125.01, 130. 01, 130.13, 132.41, 132.55, 146.68, 146.76, 176.54

^{19}F NMR (376 MHz, DEUTERIUM OXIDE) Shift ppm -62.87, -62.72

Met-FNBT

(2-nitro-4-(trifluoromethyl)phenyl)-L-methionine

mass of product: 479 mg, Yield: 94.44%, oil, $[\alpha]_D^{26}$ -120.88 (C 0.974, MeOH)

^{1}H NMR (700 MHz, DEUTERIUM OXIDE) Shift ppm 2.00 (s, 3H), 2.46-2.62 (m, 4H), 4.14-4.18 (m, 1 H), 6.85 (m, 1 H), 7.60 (m, 1 H), 8.33 (m, 1 H)

^{13}C NMR (176 MHz, DEUTERIUM OXIDE) Shift ppm 14.20, 29.46, 31.31, 57.20, 115.32, 116.85 (q, J=34.13 Hz), 123.67 (q, J=270.04 Hz), 124.81, 130.38, 132.59, 146.17, 178.47

^{19}F NMR (376 MHz, DEUTERIUM OXIDE) Shift ppm -62.80

Asn-FNBT

(2-nitro-4-(trifluoromethyl)phenyl)-L-asparagine

mass of product: 465 mg, Yield: 96.54%, mp. 193-195°C, $[\alpha]_D^{27}$ 18.03 (C 0.978, MeOH)

^{1}H NMR (700 MHz, DEUTERIUM OXIDE) Shift ppm 2.73 (dd, J-14.81 Hz, J=7.74 Hz, 1 H), 2.78 (dd, J=14.74 Hz, J=4.87 Hz, 1 H), 4.35 (dd, J=7.74, J=4.94 Hz, 1H), 6.83 (m, 1 H), 7.60 (m, 1 H), 8.34 (m, 1 H)

^{13}C NMR (176 MHz, DEUTERIUM OXIDE) Shift ppm 37.97, 55.26, 115.19, 117.17 (q, J=33.80 Hz), 124.45 (q, J=270.38 Hz), 124.84, 130.65, 132.64, 146.11, 175.47, 177.35

^{19}F NMR (376 MHz, DEUTERIUM OXIDE) Shift ppm -62.78

Pro-FNBT

(2-nitro-4-(trifluoromethyl)phenyl)-L-proline

mass of product: 454 mg, Yield: 99.44%, mp. 100-105°C, $[\alpha]_D^{27}$ -615.43 (C 1.050, MeOH)

^{1}H NMR (700 MHz, DEUTERIUM OXIDE) Shift ppm 3.08-3.19 (m, 4 H), 3.51-3.64 (m, 2 H), 4.61-4.64 (m, 1 H) 7.08 (m, 1 H), 7.70 (m, 1 H), 8.40 (m, 1 H)

^{13}C NMR (176 MHz, DEUTERIUM OXIDE) Shift ppm 33.94, 34.51, 52.69, 70,85, 115.35, 116.48 (q, J=33.91 Hz), 123.83 (q, J=270.11 Hz), 124.92, 130.21, 132.51, 146.14, 179.71

^{19}F NMR (376 MHz, DEUTERIUM OXIDE) Shift ppm –60.65

Gln-FNBT

(2-nitro-4-(trifluoromethyl)phenyl)-L-glutamine

mass of product: 494 mg, Yield: 98.19%, mp. 158-161°C, $[\alpha]_D^{27}$ -73.02 (C 0.970, MeOH)

^{1}H NMR (700 MHz, DEUTERIUM OXIDE) Shift ppm 2.03-2.10 (m, 2 H), 2.15-2.20 (m, 2 H), 4.10-4.12 (m, 1H), 6.84 (m, 1 H), 7.60 (m, 1 H), 8.35 (m, 1 H)

^{13}C NMR (176 MHz, DEUTERIUM OXIDE) Shift ppm 27.42, 31.23, 57.43, 115.38, 116.80 (q, J=36.47 Hz), 124.47 (q, J=271.71 Hz), 124.85, 130.49, 132.57, 146.08, 177.58, 178.35

^{19}F NMR (376 MHz, DEUTERIUM OXIDE) Shift ppm -62.80

Arg-FNBT

(2-nitro-4-(trifluoromethyl)phenyl)-L-arginine

mass of product: 535 mg, Yield: 98.18%, oil, $[\alpha]_D^{25}$ -125.83 (C 0.848, MeOH)

^{1}H NMR (700 MHz, DEUTERIUM OXIDE) Shift ppm 1.50-1.59 (m, 2 H), 1.60-1.66 (m, 2 H), 3.68-3.70 (m, 2 H), 4.14-4.16 (m, 1 H), 6.90 (m, 1 H), 7.67 (m, 1 H), 8.43 (m, 1 H)

^{13}C NMR (176 MHz, DEUTERIUM OXIDE) Shift ppm 24.54, 28.76, 40.59, 54.27, 115.48, 116.11 (q, J=34.46 Hz), 123.75 (q, J=262.68 Hz), 125.18, 130.49, 132.61, 146.10, 156.70, 179.10

^{19}F NMR (376 MHz, DEUTERIUM OXIDE) Shift ppm -62.80

Ser-FNBT

(2-nitro-4-(trifluoromethyl)phenyl)-L-serine

mass of product: 438 mg, Yield: 99.29%, mp. 189-191°C, $[\alpha]_D^{27}$ 65.31 (C 1.030, MeOH)

^{1}H NMR (700 MHz, DEUTERIUM OXIDE) Shift ppm 3.74-3.81 (m, 1 H), 3.86-3.93 (m, 1 H), 4.15-4.18 (m, 1 H), 6.85 (m, 1 H), 7.61 (m, 1 H), 8.36 (m, 1 H)

^{13}C NMR (176 MHz, DEUTERIUM OXIDE) Shift ppm , 56.35, 62.12, 115.50, 116.93 (q, J=34.47 Hz), 123.72 (q, J=271.71 Hz), 124.86, 130.54, 132.52, 146.41, 176.54

^{19}F NMR (376 MHz, DEUTERIUM OXIDE) Shift ppm -62.72

Thr-FNBT

(2-nitro-4-(trifluoromethyl)phenyl)-L-threonine

mass of product: 453 mg, Yield: 97.97%, mp. 165-167°C, $[\alpha]_D^{26}$ 89.18 (C 0.992, MeOH)

^{1}H NMR (700 MHz, DEUTERIUM OXIDE) Shift ppm 1.24 (d, J=6.16 Hz, 3 H), 3.99 (d, J=4.40, 1 H), 4.27 (dd, J=6.46 Hz, J=4.11 Hz, 1 H), 6.84 (m, 1 H), 7.58 (m, 1 H), 8.35 (m, 1 H)

^{13}C NMR (176 MHz, DEUTERIUM OXIDE) Shift ppm 19.61, 63.79, 68.17, 115.41, 116.90 (q, J=34.13 Hz), 123.71 (q, J=270.04 Hz), 124.88, 130.52, 132.61, 146.97, 177.34

^{19}F NMR (376 MHz, DEUTERIUM OXIDE) Shift ppm –61.28

Val-FNBT

(2-nitro-4-(trifluoromethyl)phenyl)-L-valine

mass of product: 457 mg, Yield: 99.42%, oil, $[\alpha]_D^{27}$ 90.35 (C 0.984, MeOH)

^{1}H NMR (700 MHz, DEUTERIUM OXIDE) Shift ppm 0.89 (d, J=6.94 Hz, 3 H), 0.95 (d, J=6.94 Hz, 3 H), 2.13 - 2.22 (m, 2 H), 3.86 (d, J=5.20 Hz, 1 H), 6.81 (m, 1 H), 7.57 (m, 1 H), 8.29 - 8.34 (m, 1 H)

^{13}C NMR (176 MHz, DEUTERIUM OXIDE) Shift ppm 17.73, 17.93, 30.82, 63.99, 116.62 (q, J=34.47 Hz), 122.94 (q, J=269.71 Hz), 124.75, 124.78, 130.09, 132.55, 146.92, 178.64

^{19}F NMR (376 MHz, DEUTERIUM OXIDE) Shift ppm -62.76

Trp-FNBT

(2-nitro-4-(trifluoromethyl)phenyl)-L-tryptophan

mass of product: 590 mg, Yield: 100%, mp. 119-121°C, $[\alpha]_D^{27}$ -264.71 (C 0.970, MeOH)

^{1}H NMR (700 MHz, DEUTERIUM OXIDE) Shift ppm 3.05 (m, 1 H), 3.23 (m, 1 H), 4.13 (m, 1 H), 6.19 (m, 1 H), 6.63 (br. s., 1 H, NH), 6.75 (m, 1 H), 6.80 - 6.91 (m, 1 H), 7.01 (m, 1 H), 7.11 (m, 1 H), 7.22 (m, 1 H), 7.68 (m, 1 H), 7.83 (br. s., 1 H, NH), 8.33 (m, 1 H)

^{13}C NMR (176 MHz, DEUTERIUM OXIDE) Shift ppm 28.00, 58.77, 109.73, 111.87, 115.09, 116.30 (q, J=33.46 Hz), 118.34, 119.38, 122.06, 123.51 (q, J=271.04 Hz), 124.39, 124.96, 127.03, 129.58, 131.75, 136.28, 146.03, 178.63

^{19}F NMR (376 MHz, DEUTERIUM OXIDE) Shift ppm -62.80

Tyr-FNBT

(2-nitro-4-(trifluoromethyl)phenyl)-L-tyrosine

mass of product: 553 mg, Yield: 99.51%, mp. 210-215°C, $[\alpha]_D^{27}$ -183.87 (C 0.986, MeOH)

^{1}H NMR (700 MHz, DEUTERIUM OXIDE) Shift ppm 3.03-3.15 (m, 1 H), 3.17-3.30 (m, 1 H), 4.34-4.43 (m, 1 H), 6.73 (m, 1 H), 6.81-6.88 (m, 2 H), 7.14-7.19 (m, 2 H), 7.58 (m, 1 H), 8.25 (m, 1 H)

^{13}C NMR (176 MHz, DEUTERIUM OXIDE) Shift ppm 41.49, 56.74, 115.71, 116.20 (q, J=36.81 Hz), 125.02 (q, J=259.67 Hz), 125.05, 128.64, 128.69, 129.15, 130.91, 133.55, 136.09, 168.19, 177.52

^{19}F NMR (376 MHz, DEUTERIUM OXIDE) Shift ppm -62.76

Ort-FNBT

N^2,N^5-bis(2-nitro-4-(trifluoromethyl)phenyl)-L-ornithine

mass of product: 727 mg, Yield: 95.03%, oil, $[\alpha]_D^{27}$ -129.79 (C 0.988, MeOH)

^{1}H NMR (700 MHz, DEUTERIUM OXIDE) Shift ppm 1.94-2.06 (m, 4 H), 3.95-3.98 (m, 1 H), 4.19-4.21 (m, 2 H), 6.94 (m, 2 H), 7.70 (m, 2 H), 8.48 (m, 2 H)

^{13}C NMR (176 MHz, DEUTERIUM OXIDE) Shift ppm 22.72, 27.36, 38.87, 54.10, 115.12, 115.34, 116.25 (q, J=33.12 Hz), 116.50 (q, J=34.15 Hz), 123.12 (q, J=269.88 Hz), 123.94 (q, J=270.20 Hz), 124.66, 124.86, 130. 21, 130.33, 132.55, 132.65, 146.44, 146.66, 174.19

^{19}F NMR (376 MHz, DEUTERIUM OXIDE) Shift ppm -62.88, -62.18
